# Prospective screening study of 0.5 Tesla dedicated magnetic resonance imaging for the detection of breast cancer in young, high-risk women

**DOI:** 10.1186/1472-6874-6-10

**Published:** 2006-06-26

**Authors:** Wendy S Rubinstein, Jean J Latimer, Jules H Sumkin, Michelle Huerbin, Stephen G Grant, Victor G Vogel

**Affiliations:** 1 Department of Medicine, Northwestern University Feinberg School of Medicine, Chicago, IL, USA; 2 Evanston Northwestern Healthcare Center for Medical Genetics, Evanston, IL, USA; 3 Department of Obstetrics, Gynecology and Reproductive Sciences, School of Medicine, University of Pittsburgh, Pittsburgh, PA, USA; 4 Research Institute, Magee-Womens Hospital, Pittsburgh, PA, USA; 5 University of Pittsburgh Cancer Institute, Pittsburgh, PA, USA; 6 Department of Radiology, Magee-Womens Hospital, Pittsburgh, PA, USA; 7 Department of Environmental and Occupational Health, Graduate School of Public Health, University of Pittsburgh, Pittsburgh, PA, USA; 8 Department of Medicine, School of Medicine, University of Pittsburgh, Pittsburgh, PA, USA

## Abstract

**Background:**

Evidence-based screening guidelines are needed for women under 40 with a family history of breast cancer, a *BRCA1 *or *BRCA2 *mutation, or other risk factors. An accurate assessment of breast cancer risk is required to balance the benefits and risks of surveillance, yet published studies have used narrow risk assessment schemata for enrollment. Breast density limits the sensitivity of film-screen mammography but is not thought to pose a limitation to MRI, however the utility of MRI surveillance has not been specifically examined before in women with dense breasts. Also, all MRI surveillance studies yet reported have used high strength magnets that may not be practical for dedicated imaging in many breast centers. Medium strength 0.5 Tesla MRI may provide an alternative economic option for surveillance.

**Methods:**

We conducted a prospective, nonrandomized pilot study of 30 women age 25–49 years with dense breasts evaluating the addition of 0.5 Tesla MRI to conventional screening. All participants had a high quantitative breast cancer risk, defined as ≥ 3.5% over the next 5 years per the Gail or BRCAPRO models, and/or a known *BRCA1 *or *BRCA2 *germline mutation.

**Results:**

The average age at enrollment was 41.4 years and the average 5-year risk was 4.8%. Twenty-two subjects had BIRADS category 1 or 2 breast MRIs (negative or probably benign), whereas no category 4 or 5 MRIs (possibly or probably malignant) were observed. Eight subjects had BIRADS 3 results, identifying lesions that were "probably benign", yet prompting further evaluation. One of these subjects was diagnosed with a stage T1aN0M0 invasive ductal carcinoma, and later determined to be a *BRCA1 *mutation carrier.

**Conclusion:**

Using medium-strength MRI we were able to detect 1 early breast tumor that was mammographically undetectable among 30 young high-risk women with dense breasts. These results support the concept that breast MRI can enhance surveillance for young high-risk women with dense breasts, and further suggest that a medium-strength instrument is sufficient for this application. For the first time, we demonstrate the use of quantitative breast cancer risk assessment via a combination of the Gail and BRCAPRO models for enrollment in a screening trial.

## Background

The sensitivity of mammography has been observed to be lower in women <50 years of age (63–86%), compared with women ≥50 years (89–94%) [1-3]. The U.S. Preventive Services Task Force meta-analysis found that the time required to obtain a risk reduction in breast cancer mortality rates is longer for younger women [4]. The lower sensitivity of mammography in young women has been ascribed to their higher prevalence of mammographically dense breasts and, perhaps, faster tumor growth rates [1,2].

About 25% of all women have dense breast tissue, and this physiology is more common in younger women [5-9]. Indeed, dense breast tissue has been found to be an independent risk factor for breast cancer [10,11]. Screening mammography may have limited utility in women with dense breast tissue for a variety of reasons, including: similar attenuation properties of breast lesions with dense glandular tissue; more radiation scatter and therefore higher required dose; and difficulty obtaining adequate exposure without image degradation [12]. Among women in a screening study, the sensitivity of mammography varied from 80% in women with extremely fatty breasts to a mere 30% for those with extremely dense breasts, and the odds ratio of an interval tumor was 9.47 for the latter group [13].

Younger women have a lower prevalence of breast cancer, which must be balanced against the false-positive rate of screening mammography [4]. However, the risk of breast cancer in young women with specific risk factors may equal or exceed that of older women for whom screening is unequivocally recommended. About 6% of breast cancer cases in women < 50 years of age are due to germline mutations in the *BRCA1 *or *BRCA2 *breast and ovarian cancer susceptibility genes [14,15]. Carriers are at exceptionally high risk, with potential breast cancer onset as early as the third decade and cumulative risk to age 40 reaching up to 20% [16]. While prophylactic mastectomy is the most effective risk-reducing therapy, many carriers would opt for heightened surveillance instead, given a sufficient degree of confidence in the opportunity for early detection [17].

There is evidence that breast density in *BRCA1 *mutation carriers is similar to that of women in the general population [18]. Thus, resultant technical limitations of screening mammography are likely to apply to this group of relatively young high-risk women [19]. Specifically, in a study comparing mammography among 34 *BRCA1 *or *BRCA2 *mutation carriers with breast cancer vs. disease-free controls, false-negative mammography correlated independently with *BRCA1*/2 mutation, the histological feature of prominent pushing margins, and high breast density [20].

Also relevant to the management of germline mutation carriers is the involvement of the BRCA1 and BRCA2 proteins in recombination repair of ionizing radiation-induced double-strand DNA breaks [21-23]. Possible carcinogenic consequences of low-dose irradiation for young mutation carriers undergoing earlier mammography screening must be considered [21].

An adjunct to screening mammography is particularly needed for young women at high risk of breast cancer whose imaging is limited by radiographically dense breasts. Contrast-enhanced magnetic resonance imaging (MRI) of the breast is a potential surveillance approach that is highly sensitive, is not limited by radiographic density, and poses no radiation risks. The sensitivity of contrast-enhanced breast MRI for breast cancer detection has been reported to be as high as 94%–100% [24-26]. Specificities are more variable, with values ranging from 37%–97% [25-28]. Recent larger prospective screening studies conducted in high-risk cohorts confirm high sensitivities and demonstrate, for the first time, high specificities, as well [29,30].

## Methods

### Study design

This study is a prospective, nonrandomized clinical trial designed to investigate the usefulness of a prototypical midfield strength Magnetic Resonance Imaging System in a screening setting using quantitative risk assessment for eligibility. The study looked at the addition of 0.5 Tesla MRI to a screening regimen for young women at high risk of breast cancer with dense breast tissue consisting of conventional screening modalities. The study was designed as a pilot study, with an enrollment over one year of thirty women. The data presented are a summary of this pilot study.

### Patient selection and consent

Thirty women between the ages of 25 and 49, inclusively, without a personal history of invasive or non-invasive breast cancer were recruited for this study between 10/27/99 and 4/19/00 via the joint Comprehensive Breast and Cancer Genetics Programs of the University of Pittsburgh Cancer Institute and Magee-Womens Hospital of UPMC. Women were required to have a negative or benign mammographic and physical evaluation within three months of enrollment. All subjects had mammographically dense breast tissue described as "heterogeneously" or "extremely dense" according to the American College of Radiology **B**reast **I**maging **R**eporting and **D**ata **S**ystem (BIRADS) lexicon [31]. Categorization of increased breast density was first determined by report on prior conventional film-screen mammographic examination, performed within 3 months of enrollment. Mammogram films were obtained and reviewed by the lead radiologist (JHS) to verify the presence of increased breast density. The four standard American College of Radiology BIRADS breast composition patterns are: (1) almost entirely fat; (2) predominantly fat with scattered fibroglandular densities; (3) heterogeneously dense; and (4) extremely dense. Women with fatty breasts or scattered areas of density with no focal areas of concentration were not eligible for the study. Exclusion criteria included a contraindication to MRI, breast implants, mastectomy, or a history of allergic reaction to gadolinium. Quantitative risk analysis was performed on all women prior to enrollment using the Gail and BRCAPRO-based CancerGene models. Participants had a minimum 5-year breast cancer risk on either model of 3.5% and/or a known mutation in *BRCA1 *or *BRCA2*.

This study was reviewed and approved by the Magee-Womens Hospital of UPMC Institutional Review Board (MWH-99–081). The study was explained to all participants and informed consent was obtained. Genetic testing was performed following genetic counselling under protocol MWH-97-082.

### Risk analysis

Absolute 5-year breast cancer risk was determined using the Gail and BRCAPRO models [32,33], both of which have been extensively validated [34,35]. The National Cancer Institute Gail model computer program directly calculates absolute 5-year risk of invasive breast cancer using by using a baseline proportional hazards estimation and incorporating the following risk factors: age, race, number of first-degree relatives with breast cancer (maximum of 2), age at menarche, age at first live birth (or nulliparity), number of breast biopsies (with relative risk adjusted by age), and atypical hyperplasia. The BRCAPRO program is based on the Berry model [33] and utilizes Mendelian principles with Bayesian updating to calculate carrier probabilities for germline *BRCA1 *and *BRCA2 *mutations by capturing extensive family history information about female and male breast cancer, ovarian cancer, age at cancer diagnosis, current age or age of death for relatives with and without cancer, and ethnicity. Absolute 5-year risk of breast cancer was then determined as follows: (the probability of being a *BRCA1/2 *mutation carrier based on BRCAPRO) × (the yearly incidence of breast cancer in a mutation carrier, specific to the decade of life [36]) × (5 years). These calculations were performed automatically using the CancerGene program [37], freely available on the worldwide web [38].

Family history and personal risk factors were obtained through personal interview to obtain the most complete and accurate history possible. This information was collected on case report forms and entered into the models on computer. If both models resulted in a 5 year risk of ≥3.5%, the higher value was used. As per the CancerGene program, known *BRCA1 *and *BRCA2 *carriers reach the 3.5% 5 year risk threshold at ages 27 and 32 years, respectively.

All women had a negative or benign breast examination, including physical examination and mammography within 3 months prior to study enrollment. All enrolled participants received a screening MRI as described below.

### Device information

This study utilized a contrast-enhanced magnetic resonance imaging system produced by Aurora Imaging Technology, Wilmington, MA. The system involves a 0.5 Tesla magnet with gadolinium as a contrast agent. The Aurora system is a dedicated system specifically designed for breast imaging.

The 0.5T MRI uses local volume transmit/receive coils and a gradient strength of 1 G/cm. The RF system consists of a wide band receiver operating at 21 MHz. There is 0.3 kwatts RF power. Images can be viewed at either a workstation or by film generated from an interface with a laser camera.

### Magnetic resonance imaging

Patients were scanned in the prone position and placed into the magnet feet first. Images were acquired in the axial plane with both breasts imaged simultaneously. The field of view was variable from 8–46 cm transverse and 20 cm axial. Standard 2D and 3D gradient echo and spin echo sequences were used. The dynamic, contrast-enhanced series was 3D. The matrix was 256 × 256 × 64 (x, y, z). The first sequence was non-contrast: T1 – Weighted GE, TR = 14 ms and TE = 6.0 ms, giving 64 2.0 mm thick slices, with an in-plane resolution of 1.4 mm × 1.4 mm. After completion of the non-contrast scan, intravenous gadolinium was administered at a dose of 0.1 mmol/kg of body weight at approximately 1.0 cc/sec. Post-contrast axial imaging was performed immediately, followed by a second scan approximately 4 minutes later for delayed imaging. Total imaging time is approximately 4 minutes per sequence or 12 minutes for actual imaging (this relatively long time was not inconsistent with protocols during the time of the study). Immediate and delayed subtraction views were generated by computerized subtraction of the pre-contrast image from both the immediate and delayed post-contrast images. This post-imaging subtraction was used as the method of fat suppression, i.e., no active fat suppression was used. Regions of interest (ROIs) were placed on enhancing lesions of concern and three point time dependent intensity curves were generated for each region.

Lesions were characterized using both morphologic and kinetic criteria similar to the manner described [39,40]. An MR BIRADS category was assigned ranging from 1 (negative), 2 (benign), 3 (probably benign) 4 (possibly malignant), to 5 (probably malignant). Morphology was described according to the lexicon developed by the MRI working group, which was available to us prior to the final publication date [41]. Criteria used to evaluate and rate MRI-detected lesions were those published by Schnall *et al*. [42] combined with kinetic information. Kinetics were described by visually comparing the immediate post contrast images to the delayed post contrast images. Lesions were scored as early enhancement and early washout, early enhancement and delayed washout, or delayed enhancement. In a manner similar to Hylton *et al*. [43], lesions were considered suspicious if they had either morphologic or kinetic features suspicious for malignancy (rapid wash-in and washout). In addition, lesions graded as BIRADS 3 by either morphologic or kinetic criteria were offered further evaluation using ultrasound. Ultrasound results confirmed the MRI findings, but otherwise added no new information.

### Results

Thirty women were enrolled in the study. The average age at enrollment was 41.1 years. The average 5-year breast cancer risk at enrollment, using either the Gail model or a BRCAPRO-based cancer risk model, was 4.8%. The Gail model was actually only used to establish eligibility in 7 cases, whereas the BRCAPRO-based model was used in majority of cases, 23.

**Figure 1:**
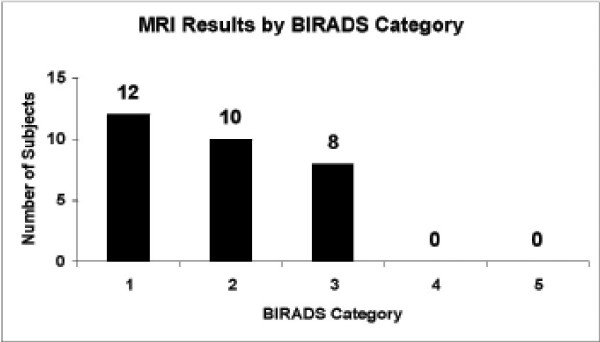


MRI results classified according to BIRADS category are shown in Figure 1 No results were observed in BIRADS categories 4 or 5. Subjects with MRI results of BIRADS 1 or 2 received no additional breast evaluations. Subjects with BIRADS 3 results were evaluated as summarized in Table 1. Follow-up involved invasive procedures in 4 of these 8 subjects. Among the 8 patients with BIRADS 3 results, one, subject 006, was diagnosed with stage I invasive ductal carcinoma.

**Table 1:** Post-MRI evaluation procedures for the BIRADS category 3 results

Procedure Type	Invasive	Non-Invasive
Short-Term (6 month) Clinical Follow-Up		1
Ultrasound with Normal Results		3
Ultrasound with Cyst Aspiration	1	
Ultrasound with Fine Needle Aspiration	1	
Ultrasound with Core Biopsy	2	

Total Patients:	**4**	**4**

**Figure 2:**
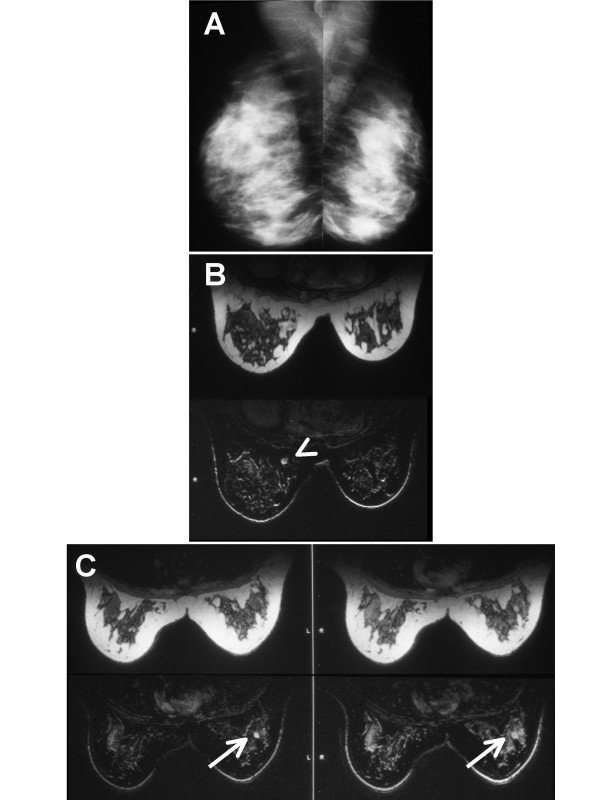
**Imaging of subject 006. **A) Subject 006 pre-MRI mammogram demonstrating heterogeneously dense breast tissue. There is no evidence of a cancerous lesion. B) Pre-contrast MR image showing an approximately 1.5 cm, smooth, round lesion in the right breast just above the nipple level medial and close to the chest wall (arrowhead). Core biopsy of this lesion demonstrated benign pathology, specifically, fibrosis with focal ductal epithelial hyperplasia [44]. C) Post-contrast MR images showing a small (approximately 1 cm), round, well-delineated enhancing mass (arrow) in the left breast at the 1:00 position. This mass was seen on both the initial delay after contrast injection (left) and the delayed contrast enhanced subtraction images (right). Core biopsy of this lesion indicated infiltrating ductal carcinoma, which was confirmed after removal via modified radical mastectomy [44].

Imaging results for subject 006 are given in Figure 2 (for a full clinical description of this patient, as well as live-cell analysis of her breast tissue for indices of proliferation, differentiation and genomic instability, see [44]). Her mammogram is depicted in Figure 2A, showing heterogeneously dense breast tissue. The MR images for this patient are shown in Figures 2B and 2C. In the upper-outer left breast there was a small (approximately 1 cm), round, well-demarcated enhancing lesion, seen on both the initial delay after contrast injection and the delayed contrast enhanced subtraction images. This lesion appeared to accumulate contrast to a greater extent on the delayed subtraction images with an additional lesion adjacent to the first. In the right breast just above the nipple level medial and close to the chest wall an additional lesion was seen in the pre-contrast image. This lesion was approximately 1.5 cm, smooth and round. Core biopsy of the left breast revealed infiltrating ductal carcinoma in 2 of 5 core fragments; high nuclear grade, with no lymphatic invasion seen. The core biopsy of the right breast demonstrated benign pathology, specifically, fibrosis with focal ductal epithelial hyperplasia. The patient chose to undergo left modified radical mastectomy with left axillary lymph node dissection and contralateral prophylactic total mastectomy because of her genetic risk status. Final pathology in the left breast was consistent with the imaging and core biopsy in size and description. Tumor size was 8 mm in greatest dimension, nuclear grade III, ER/PR and Her2/neu negative, and the nodal status (0/4) was negative (stage T1aN0M0). This subject represented 3.3% of the sampled high risk population and 12.5% of the population with "probably benign" MRIs.

Interim follow-up was required for 8 of 30 (27%) subjects, but only 4 (13%) underwent an invasive procedure. Clinical follow up was available on all 29 disease-free subjects at least one year beyond the performance of the study MRI, which is the standard length of follow up to exclude occult cancer, and no subjects developed breast carcinoma. Thus, the patients in BIRADS categories 2 and 3 likely did not harbor occult disease.

## Discussion

Without data on cancer-specific mortality reduction, the decision to employ breast MRI surveillance rests heavily on other parameters, including test performance characteristics, cost, and methods of maximizing benefit vs. risk. Recent prospective studies have provided firmer data indicating that high sensitivity can be achieved with breast MRI without greatly sacrificing specificity. While reduction in cancer-specific mortality is the gold standard for surveillance tools, there is a pressing need to supplement mammography in high-risk women, particularly for *BRCA1 *and *BRCA2 *mutation carriers who are diagnosed with a high rate of interval tumors, roughly 50% [45]. Breast MRI results in a lower rate of interval tumors while circumventing the limitations of surveillance for women with dense breasts. However, for whom should breast MRI surveillance be employed and what are acceptable costs?

### Medium vs. high field strength MRI

Despite the promise of breast MRI, there are still some issues that must be resolved prior to its use as a standard adjunct method of breast cancer surveillance. There is not yet a standard method in place for such imaging, and no consensus on how to best interpret lesions detected by MRI, and whether or when to biopsy lesions detected by MRI alone [see ref. 31]. Additionally, the high cost of breast MRI is severely limiting. Not only is there a large cost associated with the purchase of a high field strength magnet, but also there are additional costs for housing and maintaining the unit. The use of medium field strength magnets has been criticized for their low signal-to-noise ratio (SNR) per sampling time. However, the use of a medium field strength magnet, such as a 0.5 Tesla, would come at significant cost savings of up to 1/3 that of a higher field strength MRI system. Our study provides preliminary evidence that a medium field strength breast MRI system can be effectively used for high-risk surveillance.

There is evidence that the use of a medium field strength breast MRI system is comparable to a 1.5 T system. In a study published by Kuhl *et al*. [46], MRI was performed on a midfield system without loss of sensitivity as compared to a high field system. The study looked at 42 patients imaged on both a 0.5 T and 1.5 T MRI, finding that the image quality was comparable, and, with certain compensations, the 0.5 T system was more sensitive than the larger 1.5 T MRI. In a second study, Kuhl *et al*. [47] imaged 40 patients with nodular lesions using both 0.5 T and 1.5 T field strength units to determine if the two systems were comparable in selecting benign vs. malignant lesions. Malignant lesions and fibroadenomas demonstrated a similar enhancement uptake pattern on both systems. A rapid wash-out of contrast was seen only in malignant lesions, which appeared 10 times more frequently using the 0.5 T system as compared with 1.5 T MRI.

The appearance of T1-weighted gradient echo images generated from a contrast study depends on the SNR, the contrast-to-noise ratio (CNR) and the contrast agent used. The SNR is a function of the magnetic field strength, magnet shim, flip angle, voxel size, receiver gain, RF coil and image processing parameters. Magnetic field strength directly affects the SNR and the spin relaxation properties of the tissue. The SNR is linear with field strength--everything else being equal, 1.5 T magnets produce images with three times the SNR of images from 0.5 T magnets. In addition, the T1 of a given tissue/sample type is larger at higher field strengths. This implies that at a higher field strength, the spin relaxation of a given tissue/sample may be changed more that the same tissue/sample at a lower strength and thus may increase the difference observed in pre- and post-contrast images.

The CNR is a measure of the average intensity of an object compared to the average intensity of the noise floor for a given object and pulse sequence. The CNR is a function of the relation of the pulse sequence timing parameters (e.g., TE, TR) to the spin relaxation properties (e.g., T1, T2) of the object. Because the contrast agent changes the spin relaxation properties, it changes the CNR of the image. Above a minimum SNR threshold, the ability to detect a lesion using MRI is a function of the change in CNR of pre- and post-contrast images and the voxel size acquired. If the configuration of a 1.5T MRI scanner and a 0.5T MRI scanner is such that both are above the minimum level of SNR, have comparable CNR changes after adding a contrast agent, and have identical voxel sizes and scan durations, the ability to detect lesions is similar.

Our study demonstrates that medium field strength MRI can detect tumors that have been missed by conventional screening mammography. There are, however, certainly limitations to this initial study, including its small size; up to 3 months time differential between screening mammogram and MRI; and lack of longitudinal follow up. No conclusions can be drawn regarding the overall sensitivity or specificity of screening with the 0.5 T MRI.

### Screening guidelines for young high-risk women

There has been a dearth of evidence-based screening guidelines for women age < 40 with a family history of breast cancer, a *BRCA1 *or *BRCA2 *mutation, or other risk factors, largely because of the lack of randomized, controlled trials to inform the development of such guidelines [48-50].

As evidence accumulates regarding the efficacy of bilateral prophylactic mastectomy, data regarding the efficacy of screening is of paramount importance so that women can make informed choices. In a large screening study of 251 mutation carriers, a high rate of interval tumors found on breast self-examination led to the suggestion that more frequent (i.e. semi-annual) mammography should be considered, particularly in younger women [51]. However, among women age 40–69, the estimated cumulative risk of a false positive result after 10 mammograms is 49% [52], resulting in additional visits, diagnostic tests, invasive procedures, morbidity, cost, and anxiety. Furthermore, there is evidence that false-positive rates are higher in younger women [53-55]. Ultimately, the sequelae of screening (such as biopsies) can spur the decision to undergo prophylactic surgery, giving one pause about recommending more frequent screening.

The variations in sensitivity and specificity for breast MRI exist for several reasons, including technical factors [56], interpretation criteria [28,30,38], patient selection, concomitant use of conventional imaging, and the level of pathologic verification of the abnormalities detected. The large disparity in specificities results from a variety of technical factors. For instance, the lowest reported specificity was calculated without the use of morphologic features or quantification of enhancement, and certain high-risk lesions, such as atypical ductal hyperplasia, were considered as false positives [24].

All high-risk MRI screening studies reported thus far have used high field-strength magnets. Stoutjesdijk *et al*. [57] found that for the indeterminate BIRADS score of 3, the sensitivity of breast MRI was 100% with a specificity of 93% (95% confidence interval = 90%–96%) and a positive predictive value of 43%. Warner *et al*. [58] reported a sensitivity of 100% and noted that all four false negative mammograms had a BIRADS score of 1. In this study, increased breast density appeared to contribute to the poor sensitivity of mammography. An update of this study reporting findings on 236 Canadian women aged 25 to 65 years with *BRCA1 *or *BRCA2 *mutations found a specificity of 95.4% based on biopsy of BIRADS level 4 and 5 lesions, but did not take into account the effect of breast density, nor the non-biopsy interventions engendered by level 3 lesions [30]. High-risk women may be particularly susceptible to the emotional turmoil triggered by a diagnostic workup for breast cancer, considering their high-risk family histories.

The largest breast MRI surveillance study reported to date, based on 1909 eligible women including 358 germ-line mutation carriers, found a specificity of 89.8% for workup of level 3, 4, and 5 lesions [29]. However, this study did not examine possible differences in test performance among women at varying levels of risk, nor was breast density taken into account. While a lower limit of 15% lifetime risk constituted study eligibility, it remains unclear whether this risk level merits high-risk surveillance, particularly in women with average mammographic breast density. Our ongoing studies of surveillance screening with medium field strength MRI have shown that the false-positive rate is three-fold lower than that of mammography [59].

## Conclusion

### Toward quantitative balancing of risks and benefits in surveillance

We contend that the benefits of breast cancer surveillance cannot be satisfactorily balanced against the risks of screening without an accurate assessment of absolute breast cancer risk. We report for the first time the use of quantitative breast cancer risk assessment for use in enrollment in a breast cancer screening pilot study by combining both the established Gail model and a purely genetic model which estimates *BRCA1/2 *mutation risk via BRCAPRO. Kriege *et al*. [29] used the Claus model [60] for risk stratification, but this model has not been as extensively validated as either the Gail or BRCAPRO models. In particular, omission of family history of ovarian cancer in the Gail and Claus models is one factor that can lead to underprediction of breast cancer risk; this constitutes a serious limitation to using either model alone [61]. The Gail model identifies at least three types breast cancer risk factors: genetic risk associated with family history, risk based on lifestyle factors associated with hormonal effects, and risk associated with suspicious breast pathologies, regardless of the basis of this risk. Genetic analysis of the subjects who developed breast cancer in the NSABP P-1 Breast Cancer Prevention Trial indicates that the Gail model inefficiently identifies *BRCA1 *and *BRCA2 *mutation carriers, since only 6.6% (19/288) of that high-risk population carried such mutations [62].

Supplementation of the Gail model with the BRCAPRO model allows for a much more efficient ascertainment of women at high risk for breast cancer specifically due to the possibility that they carry a mutant *BRCA1 *or *BRCA2 *gene, based on our accumulated knowledge of hereditary breast-ovarian cancer syndrome. Since 23 of our 30 subjects were enrolled based on their BRCAPRO risk rather than their risk based on the Gail model, this group represents a rather unique "high-risk" population, with perhaps more in common with studies in *BRCA1/2 *carriers such as that of Warner *et al*. [30] than previous populations enrolled through the exclusive use of the Claus model. We are the first to incorporate a quantitative cancer risk component based on genetic risk as assessed by BRCAPRO, without direct confirmation by gene testing. This is important because some subjects with *BRCA1 *or *BRCA2 *mutations may be reluctant to enroll in a trial restricted to mutation carriers, since their genetic status would be known by virtue of their participation. Enrollment according to quantitative risk can allow women who are not yet prepared for genetic testing to participate in a high-risk surveillance trial.

We have advanced the rationale for quantitative risk assessment in chemoprevention trials, with the advantages of improved power using a smaller study population, shorter study duration and lower cost [63]. The same rationale applies to surveillance trials. In addition, subjects at higher risk of breast cancer have a greater ratio of benefit vs. risk than average-risk women since, if the surveillance modality is efficacious, they have a greater opportunity for early detection. For trials that seek to measure specificity, breast cancer events are not crucial to determining the required sample size, yet for ethical reasons, subjects who stand to benefit the most ought to be preferentially studied. Ours is the first study to concentrate on high-risk women with mammographically dense breast tissue; the poorer sensitivity of mammography in this group should contribute to an especially high ratio of benefit vs. risk.

The concomitant use of the Gail and BRCAPRO-based models allows for the identification of a broad array of high-risk women [64]. The report of the Working Groups on Breast MRI advises that "a careful analysis of the woman's actual risk for breast cancer" be done when considering the appropriateness of screening MRI [65]. They urge the development of partnerships with high-risk clinics and/or clinicians with significant experience with high-risk women. Ultimately, since the conduct of randomized, controlled trials in high-risk women faces numerous challenges, medical decision making models may be useful for balancing the benefits and risks using such parameters as age, quantitative breast cancer risk, and breast density.

## Competing interests

The author(s) declare that they have no competing interests.

## Authors' contributions

WSR designed the study, recruited and consented the patients, obtained funding, and drafted the manuscript. JJL and SGG contributed to the study evaluation and drafted the manuscript. JHS was responsible for the performance and interpretation of the mammography and MRI. MH was the research coordinator and helped design the study. VGV participated in the study design and data interpretation. All authors read and approved the final manuscript.

## Pre-publication history

The pre-publication history for this paper can be accessed here:


